# Supplementary material for: Examining Perceived Stress and Coping Strategies of University Students during COVID-19: A Cross-Sectional Study in Jordan
Source: Int J Environ Res Public Health. 2022 Jul 27;19(15):9154. doi: 10.3390/ijerph19159154 (PMC9368044; doi:10.3390/ijerph19159154)
Supplement: Supplementary file 1 [file ijerph-19-09154-s001.zip › ijerph-1822616-supplementary.pdf]

Examining perceived stress and coping strategies of university students during COVID-19: A cross-sectional study in Jordan

This questionnaire examines COVID-19's impact on Jordanian business students' academic performance during the lockdown. Business undergraduates, graduates or recent graduates can fill out this survey. All responses will be analysed to conduct research. Please note that participation in this survey is voluntary and anonymous. Answering this questionnaire indicates the consent to participate in this study.

* Required

1. Gender *

Mark only one oval.

- Male
- Female

2. Age *

Mark only one oval.

- 18-20
- 21-23
- 24-26
- 27 and older

3. Your academic year *

Mark only one oval.

- 1st year
- 2nd year
- 3rd year
- 4th year
- Recently Graduated
- Other: ______________________

4. Place of residency *

Mark only one oval.

- Amman
- Irbid
- Zarqa
- Kerak
- Salt
- Mu'tah
- Ajloun
- Aqaba
- Jerash
- Ma'an
- Tafilah
- Mafraq
- Madaba

5. Degree level *

Mark only one oval.

- Undergraduate
- Postgraduate

6. University *

Mark only one oval.

- Balqa Applied University- Ajloun College
- Ajloun National University
- Al-Ahliyya Amman University
- Al-Isra University
- Al-Zaytoonah University of Jordan
- Amman Arab University
- Applied Science Private University
- Arab Academy for Banking and Financial Sciences
- Arab Open University
- Columbia University: Amman Branch
- German-Jordanian University: Jabal-Amman Branch
- German-Jordanian University: Almushaqar campus/Main Campus
- Hussein Technical University
- Jordan Academy for Maritime Studies
- Jordan Academy of Music
- Jordan Institute of Banking Studies
- Jordan Media Institute
- Luminus Technical University College
- Middle East University
- National University College of Technology
- Petra University

- Philadelphia University
- Princess Sumaya University for Technology
- Queen Noor Civil Aviation Technical College
- Tafilla Technical University
- The World Islamic Science & Education University (W.I.S.E)
- University of Jordan
- Aqaba University of Technology (2011)
- Institute of Banking Studies: Aqaba Branch
- Aqaba Campus of the University of Jordan
- Balqa Applied University (Salt)
- Irbid National University
- Jordan University of Science and Technology
- Luminus Technical University College - LTUC
- Institute of Banking Studies: Irbid Branch
- Yarmouk University
- Jadara University
- Jerash Private University
- Mutah University (in Mu'tah)
- Balqa Applied University (kerak)
- Al-Hussein Bin Talal University
- Balqa Applied University- College of Agriculture (in Shoubak)
- Balqa Applied University- College of Ma'an (in Ma'an)
- American University of Madaba (AUM):Madaba Campus
- New York Institute of Technology, Madaba

- Al al-Bayt University
- Tafila Technical University
- Hashemite University
- Zarqa Private University

7. GPA *

Mark only one oval.

- <2.00
- 2.00 - 2.5
- 2.6 - 3.50
- 3.6 - 4.0

8. During the lockdown, what electronic device did you use to study online? *

Mark only one oval.

- Laptop,
- PC
- Smartphone
- Tablet

9. What online learning tool did you use during a lockdown? *

Mark only one oval.

- Zoom
- Microsoft Teams
- Skype
- Web Whiteboard
- Edmodo
- Google Meet
- Social networks
- WhatsApp
- Others

10. What virtual learning tools did you use during the lockdown? *

Mark only one oval.

- University platforms
- Online classes
- educational websites
- YouTube videos
- E. Books
- Educational application
- PDF lectures
- Others

11. What is the average number of hours you spent online during a lockdown? *

Mark only one oval.

- 1 hour/ day
- 2 hours/ day
- 3 hours/ day
- 4 hours/ day
- 5 hours/ day
- 6 hours/ day
- 7 hours/ day
- 8 hours/ day
- 9 hours/ day
- 10 hours or more/ day

Examining perceived stress and coping strategies of university students during COVID-19: A cross-sectional study in Jordan

12. GPA *

Mark only one oval.

- <2.00
- 2.00 - 2.5
- 2.5 - 3.00
- 3.5 - 4.0

13. During COVID-19, how do you rate online education in practical lessons? *

Mark only one oval.

	1	2	3	4	5	6	7	8	9	10	
Bad											Excellent

14. In the COVID-19 pandemic, how do you rate online education (understanding course contents through an online educational platform)? *

Mark only one oval.

	1	2	3	4	5	6	7	8	9	10	
Bad											Excellent

15. How much of your academic future is at risk due to COVID-19? *

Mark only one oval.

	1	2	3	4	5	6	7	8	9	10	
Not at all at risk											Extremely at risk

16. Perceived Stress *

Mark only one oval per row.

	Never	Almost never	Sometimes	Fairly often	Very often
**How often have you felt powerless to control important events in your life?**					
**How often did you feel nervous or stressed?**					
**How often have you felt that you could not cope with everything you had to do?**					
**How often have you been angered by situations that were beyond your control?**					
**How often have you felt you could not overcome difficulties that accumulated so high?**					

17. Academic stress *

Mark only one oval per row.

	Not affected	Slightly affected	Moderately affected	Considerably affected	Greatly affected
**How did the COVID-19 pandemic affect your study/ research?**					
**My performance in exams has decreased during COVID19**					
**Learning motivation**					
**Online education during COVID-19 has increased in Instructional packages**					

18. Financial stress *

Mark only one oval per row.

	Never	Rarely	Sometimes	Often	always
**Available Suitable home learning space**					
**My budgeting was impacted by the pandemic**					
**I could not pay education fees**					
**The household's financial well-being suffered as a result**					

19. Behavior stress *

Mark only one oval per row.

	Never	Rarely	Sometimes	Most of the time	Almost constantly
**How often are you completing risk management behaviors? (Washing hands, social distancing staying inside, etc.)**	◯	◯	◯	◯	◯
**Studying online increases distraction in your environment**	◯	◯	◯	◯	◯
**Inability to interact with others or to communicate with them effectively**	◯	◯	◯	◯	◯
**In light of COVID-19, how likely are you to reduce (or withdraw) from your courses?**	◯	◯	◯	◯	◯

20. Emotional well-being: coping *

Mark only one oval per row.

	Not well at all	Slightly well	Moderately well	very well	Extremely well
**Compared to those around you (e.g., family, friends, and co-workers), how well do you feel you are coping with disruptions in your life caused by COVID-19**	◯	◯	◯	◯	◯

This content is neither created nor endorsed by Google.

Google Forms
